# Polymorphisms of HLA-DRB1, -DQA1 and -DQB1 in Inhabitants of Astana, the Capital City of Kazakhstan

**DOI:** 10.1371/journal.pone.0115265

**Published:** 2014-12-22

**Authors:** Alexandr B. Kuranov, Mikhail N. Vavilov, Gulshara Zh. Abildinova, Ainur R. Akilzhanova, Aisha N. Iskakova, Elena V. Zholdybayeva, Margarita N. Boldyreva, Claudia A. Müller, Kuvat T. Momynaliev

**Affiliations:** 1 Medical University Clinic, Tübingen, Germany; 2 Chelyabinsk Regional Blood Centre, Chelyabinsk, Russian Federation; 3 National Research Center for Maternal and Child Health, Astana, Kazakhstan; 4 National Center for Biotechnology, Astana, Kazakhstan; 5 NRC Institute of Immunology FMBA of Russia, Moscow, Russian Federation; Instituto de Higiene e Medicina Tropical, Portugal

## Abstract

**Background:**

Kazakhstan has been inhabited by different populations, such as the Kazakh, Kyrgyz, Uzbek and others. Here we investigate allelic and haplotypic polymorphisms of human leukocyte antigen (HLA) genes at DRB1, DQA1 and DQB1 loci in the Kazakh ethnic group, and their genetic relationship between world populations.

**Methodology/Principal Findings:**

A total of 157 unrelated Kazakh ethnic individuals from Astana were genotyped using sequence based typing (SBT-Method) for HLA-DRB1, -DQA1 and -DQB1 loci. Allele frequencies, neighbor-joining method, and multidimensional scaling analysis have been obtained for comparison with other world populations. Statistical analyses were performed using Arlequin v3.11. Applying the software PAST v. 2.17 the resulting genetic distance matrix was used for a multidimensional scaling analysis (MDS). Respectively 37, 17 and 19 alleles were observed at HLA-DRB1, -DQA1 and -DQB1 loci. The most frequent alleles were HLA-DRB1*07:01 (13.1%), HLA-DQA1*03:01 (13.1%) and HLA-DQB1*03:01 (17.6%). In the observed group of Kazakhs DRB1*07:01-DQA1*02:01-DQB1*02:01 (8.0%) was the most common three loci haplotype. DRB1*10:01-DQB1*05:01 showed the strongest linkage disequilibrium. The Kazakh population shows genetic kinship with the Kazakhs from China, Uyghurs, Mongolians, Todzhinians, Tuvinians and as well as with other Siberians and Asians.

**Conclusions/Significance:**

The HLA-DRB1, -DQA1and -DQB1 loci are highly polymorphic in the Kazakh population, and this population has the closest relationship with other Asian and Siberian populations.

## Introduction

**Figure 1:**
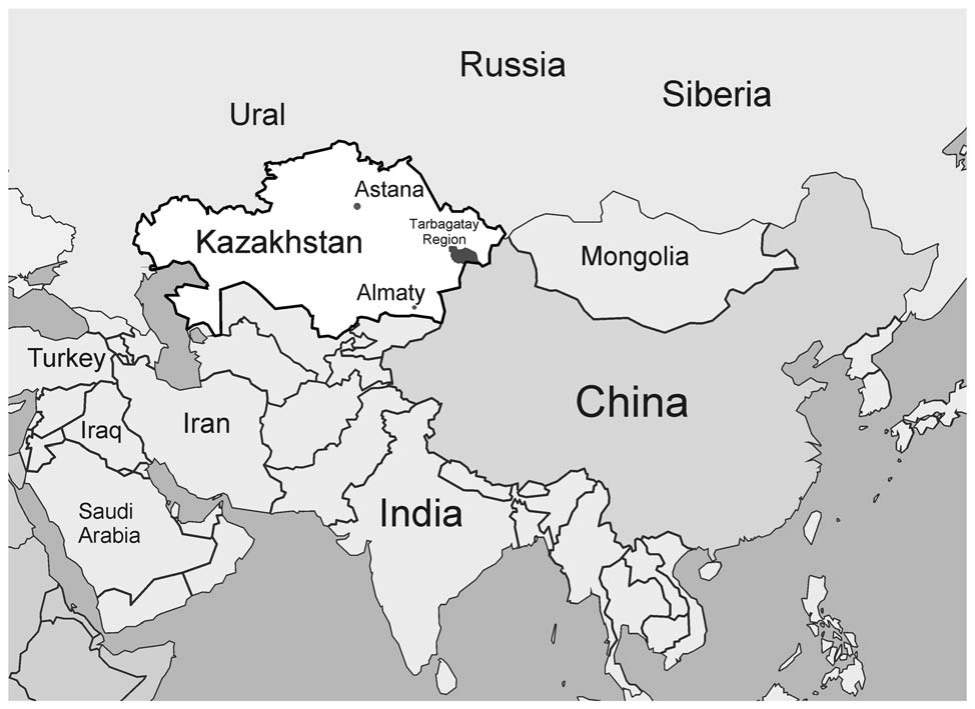
Territory of the Republic of Kazakhstan.

Kazakh Khanate (Kazakhskoye khanstvo) was established as the first Kazakh state in 1456 (1465/66) and was located in the territory of the present day Republic of Kazakhstan (Fig 1). This country is located in Central Asia, which lies on the border of Europe and Asia. This area was the intersection of many transport routes; west to Europe, east to Asia and Siberia. So Kazakhstan is located in an area where the population is characterized by different languages, religions and cultures. Many ancient tribes were involved in the formation of the Kazakhs. Anthropologists believe that the initial formation of a distinct Kazakh population began in the first millennium AD, and is considered an ancient Kazakh anthropological type with distinct features from those of European or Mediterranean anthropological types. In subsequent periods, during the Mongol invasions, an intensive mixing, resulted in Kazakhs acquiring mongolian traits [1]. Subsequently, the modern Kazakh population was formed from many different ancestor groups including Turkic tribes (Kipchaks, Argyns, Khazars etc.), Turko-Mongol tribes (Dughlat, Jalayir, Naimans etc.), and other Asian tribes. Even though Kazakhstan is basically characterized as a polyethnic country, a major section of the population (more than 60%) are Kazakhs. Kazakhs are a Turkic-speaking people, living in several Central Asian countries including Kazakhstan, Usbekistan, Kyrgyzstan, Russia, Mongolia, and China etc.

The targets of our study were: HLA-Typing of HLA-DRB1, DQA1 and DQB1 loci in the Kazakh population living in the new capital city of Kazakhstan; investigation of allele and haplotype frequencies in relation to HLA-DRB1 polymorphism; and comparisons with other world populations with different historical backgrounds in order to further understand the genetic background and the origin of the Kazakh population. The HLA class I and II are recognized as essential components of the immune response with a high polymorphism. More than 10,000 alleles are in the latest version 3.15. (2013-07) of the IMGT/HLA Database, which provides a specialised database for sequences of the HLA-complex and official sequences for the WHO nomenclature Committee for factors of the HLA system [2].

Results of the HLA-study in populations with different ethnic backgrounds are the basis for development in several areas of clinical transplantation, diagnostics, forensics and can be considered as an anthropological guide. This is a prerequisite for research of HLA-diversity in the population of Kazakhstan. The distribution of specific HLA genes in representatives of a healthy group can be used as reference markers to search for genetic predispositions of various diseases in the Kazakh ethnic group. This could serve as a theoretical basis for clinical transplantation and to find donors of allogeneic bone marrow from the same ethnic group. In our study, we focused on the study of HLA-DRB1 alleles in the Kazakh population living in Astana. There were also other classic distributions of alleles in the HLA class II. The aim of this work was to investigate HLA-genetic heterogeneity among Kazakhs by studying allele- and haplotype frequencies in relation to the HLA-DRB1 locus based on its high polymorphism. We hypothesized that, relying on the use of HLA-distribution, the origin of the Kazakh population can be determined.

## Materials and Methods

### Ethical Statement

This project was approved by the Ethics Committee of the National Center for Biotechnology, Kazakhstan (№ 10, 14.02.2010). The ethics committee approved the informed consent for this study. The investigation was conducted in accordance with humane and ethical research principles of National Center for Biotechnology. All 314 study participants completed a questionnaire requiring them to be healthy, provided informed consent, and included information regarding family history, lineage, etc. We confirm in our consent statement that consent was provided by 314 healthy individuals.

### Study Population

HLA typing and population studies were performed on 157 Kazakh individuals, 69 male and 88 female, living in Astana during 2010–2011. All individuals included in the present study were unrelated, without any sign of clinically diagnosed diseases, and randomly selected from different regions of Kazakhstan. The study participants consisted of representatives of Kazakh nationality only, and were classified as mono-ethnic according to their phenotype characteristics and family origin [1]. In 2010, 157 blood samples were collected from healthy adult individuals, 67 men and 90 women, during an expedition to Tarbagatay, East Kazakhstan for HLA-DRB1 typing. With the help of the local Medical University of Semipalatinsk, randomly selected healthy, unrelated Kazakh people from the East Kazakhstan region, Tarbagatay district (v.Karasu, v.Kabanbay, v. Akzhar) were chosen. All of these individuals' ancestors were born and lived in the Tarbagatay region of East Kazakhstan for at least three generations. Geographical location of these regions is represented on the map (Fig. 1). The following populations from different geographic regions were used in this study – Western European populations: Austrians [3], English [4], German [3], French [5], Italians (North Italy) [3], Netherlands [3], Portuguese [3], Spanish (Madrid) [6], Spanish (Granada) [7]; Eastern European populations: Albanians [8], Bashkirs [9], Belarussian [10], Bulgarian [11], Chuvashians [12], Polish [13], Russian (Ural) [9], Russian (North-West) [14]), Serbs [3], Slovaks [15], Ukranian [10]; Mediteranean populations: Armenians [16], Cretans [17], Georgians [18], Greece [3], Italians (South Italy) [3], Jews Ashkenazi [19], Arabs [20], Kurds [21], Lebanese [22], Macedonians [23], Palestinians [24]; Scandinavian populations: Finnish [3], Khanty-Mansi [25], Komis [26], Norway [27], Pomors [28], Saami [28], Swedish [29]; Siberian populations; Aleuts [30], Chukchi [31], Evenks (2 populations) [25], [31], Ket [31], Koryaks [31], Buryat [25], Nedigal [25], Nentsy [28], Nivkhs [31], Nganasan [32], Todzhinians [25], Tofalar [25], Tuva (2 populations) [25], [33], Udegeys [25], Ulchi [33]; Asian populations; Mongolian [3], Han Chinese [34], Japanese [35], Koreans [36], Kazakh (China) [37], Taiwanese [3], Uyghurs [38], Malay [39], Thai [3], Vietnamese [40], Turkish [41]; American populations: Argentine [3], Mazatecans [42], Ache [43], Eskimos (2 populations) [31], [44], Yupik [45].

### HLA Genotyping

For HLA-DRB1, -DQB1 and -DQA1 loci, allele polymorphisms were typed using the sequence-based typing (SBT) method. Genomic DNA from whole blood samples was extracted using a DNA Purification Kit (PROMEGA, Madison, WI) according to the manufacturer's protocol. The concentration of DNA was 50–100 ng/ml, with the purity of the extracted DNA ranging from a 1.5 to a 1.8 OD value. PCR and sequencing were performed for exon 2 of the HLA-DRB1, -DQA1 and -DQB1 genes using the SBT-method and locus, group, and sequence-specific primers according to multiple sources [46]–[52]. The thermal cycling profile for the amplification began with initial denaturation for 5 min at 94°C, followed by 10 cycles of 30 s at 94°C, 50 s at 65°C, 20 subsequent cycles, each consisting of annealing of the primers at 62°C for 50s and an elongation and 60 s at 72°C, with a final elongation for 5 min at 72°C. Polymerase chain reaction (PCR) was performed in 50 µl reaction mixtures of 100 mM Tris–HCl (pH 8.0) 2.5 mM MgCl2, 100 mM of each dNTP, 10 pmol of each primer, 2.0 U of Taq DNA polymerase and DNA was 50 ng/ml. For amplification the 96-well thermocycler (BioRad, Hercules, CA) was used. Amplification was verified by 2% agarose gel electrophoresis. Sequencing was performed on Genetic Analyzer (Applied Biosystem, Foster City, CA) with 96-capillaries using BigDye Terminator v3.1 chemistry (Applied Biosystem). The HLA alleles were identified using international database IMGT/HLA database [53] and a program dbMHC SBT Input [54]. This typing procedure has been published (Kuranov et al., 2014).

### Statistical Analysis

Allelic frequencies of HLA-DRB1, -DQB1 and -DQA loci were estimated by the direct counting method. Allele frequencies, haplotype frequencies, neighbor-joining dendrograms and multidimensional scaling analysis were obtained for comparing Kazakhs and worldwide populations. Statistical analyses were performed using Arlequin v3.11. The resulting genetic distance matrix was used for a multidimensional scaling analysis (MDS), for two dimensions. MDS for pairwise populations was computed using allele frequencies, based on the Euclidean distance matrix [55], [56], applying the software PAST v. 2.17. The haplotype frequencies were estimated according to allele frequencies using the expectation maximization (EM) method with the Arlequin v3.11. Tests of Hardy-Weinberg equilibrium and Linkage disequilibrium (LD) were also perfomed using this software. LD (D) coefficient has been estimated for the strength of LD (>0.80 strong LD, −0.5 moderate LD, −0 weak LD) [57]. Assuming that the (D) values might show two rare alleles that were only accidentially linked to validate all D data, the statistic parameter t [58] (t values>2.0) was used to improve results [59]. Phylogenetic dendrograms were created using the neighbor-joining (NJ) method with Nei distances, applying the phylogeny program Phylip, based on allelic frequencies [60].

## Results

### HLA Polymorphisms of the HLA-DRB1, -DQA1 and -DQB1

**Figure 2:**
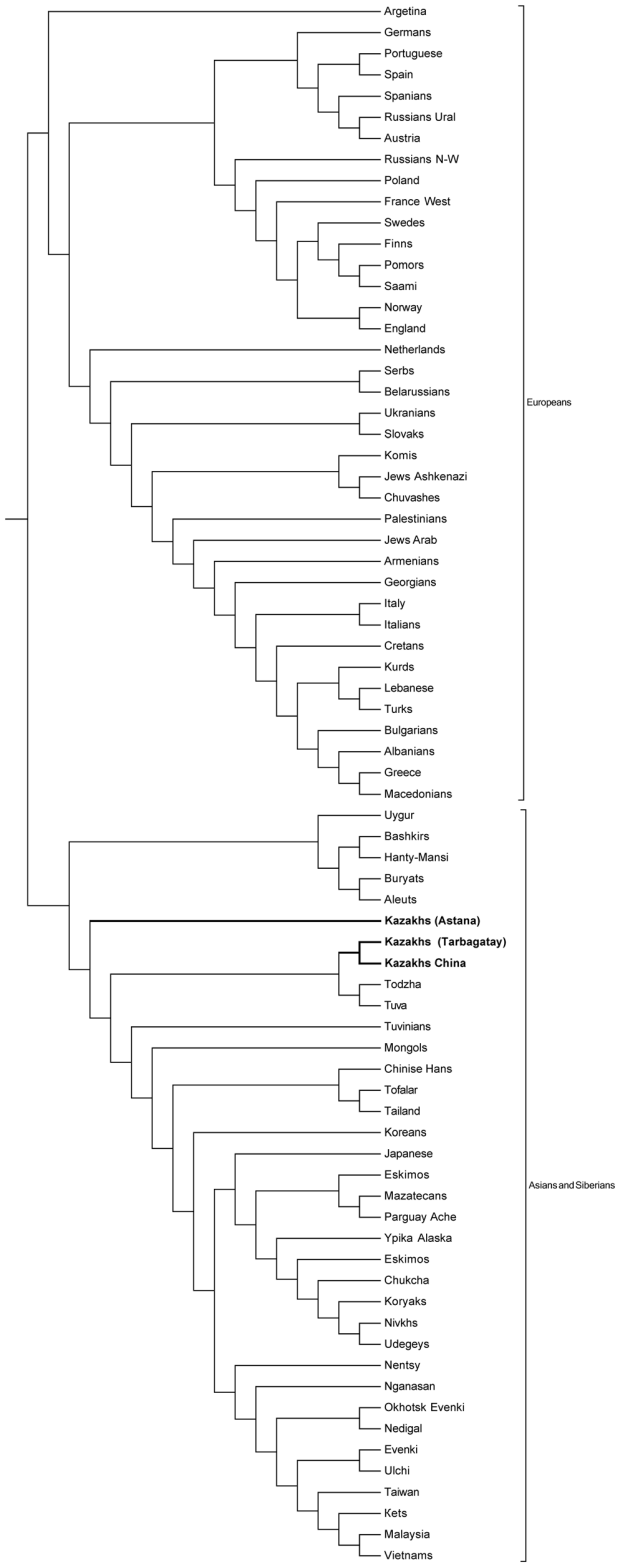
Neighbor-joining dendrogram based on HLA allele frequencies. Dendroram constructed by the neighbor-joining method showing the relationship between Kazakh populations with other populations based on the frequencies of HLA-DRB1 loc.

This first HLA-study was preferred to compare HLA-DRB1 frequencies in Kazakh population with Mediterraneans, Europeans, Scandinavians, Asians and Siberians. The HLA-DRB1 neighbor-joining dendogramm shows the Kazakh population together with Asian and Siberian populations, and separated from the European, Scandinavian and Mediterranean populations. The multidimensional scaling analysis (MDS) based on variances of the mean genetic distances were perfomed, and it was also observed here that Kazakhs clustered together with Asian and Siberian populations, however were separated from the Mediterranean, Scandinavian, European, and American populations (Fig. 2). Table 1 summarizes the HLA-DRB1, -DQB1, and DQA1 number of alleles obtained from the Kazakh population (Astana). In the 157 analyzed Kazakh individuals from Astana, 37 alleles at the HLA-DRB1 locus, 17 alleles at the HLA-DQA1 locus, and 19 alleles at the HLA-DQB1 locus were identified. The allelic frequency distributions of the HLA-DRB1, -DQA1 and -DQB1 loci in the Kazakh population of the Astana region are shown in Table 2. DRB1*07:01 and DRB1*03:01 were observed most often with a allele frequency of 13.1% and 10.0% respectively. DRB1*13:01 was detected in Kazakhs with frequency of 8.6%. Six HLA-DRB1 alleles had frequencies higher than 4% and cumulative frequency was 28.6% (DRB1*01:01, DRB1*09:01, DRB1*13:03-103, DRB1*14:01, DRB1*14:05-72 and DRB1*15:01). HLA-DQB1 is the second major HLA-locus used to investigate the formation of the Kazakh population and their common ancestors. Three HLA-DQB1 alleles: DQB1*02:01–13.6%, DQB1*03:01–17.6%, DQB1*03:04-22–11.7% present with the highest frequencies in the Kazakh population. Frequency of their occurrence in the studied population is more than 40%. At the DQA1 locus, DQA1*03:01 was found to be the most frequent (13.1%), followed by DQA1*01:02 (10.2%), DQA1*01:03 (9.6%), DQA1*02:01 (9.6%) and DQA1*05:01 (9.6%). We observed a 0.3% to 9.0% range in frequencies for the other alleles. The novel allele designations have been officially assigned by the World Health Organization Nomenclature Committee [61]. According to investigations the following 7 novel alleles were found: DRB1*03:56, DRB1*03:57, DRB1*13:102, DRB1*13:02:04, DRB1*13:103, DRB1*11:04:06 and DRB1*14:12:02 [62]. The allelic frequency distribution of the HLA-DRB1 locus in the Kazakh population of Tarbagatay region are shown in Table 3. In all, 35 alleles were identified at the HLA-DRB1 locus in the 157 individuals of the Kazakh population of Tarbagatay, the most common alleles were DRB1*13:01 (8.0%), DRB1*15:01 (7.6%), DRB1*03:01 (7.6%), DRB1*01:01 (5.4%), DRB1*13:03–102 (4.8%) and DRB1*09:01 (4.8%).

**Table 1:** Baseline data on number of alleles in the Kazakh population (Astana) and average heterozygosity.

Number of alleles per locus, observed and expected heterozygosity				
Locus	Number of Alelles	Het_obs_	Het_exp_	P-Values
HLA-DRB1	37	0.93631	0.94593	ns
HLA-DQB1	19	0.91083	0.91758	ns
HLA-DQA1	17	0.87898	0.91144	ns

**Table 2:** Allelic frequency of HLA-DRB1, -DQA1 and -DQB1 loci in the Kazakh population (Astana).

DRB1	Allelic frequency, %	DQA1	Allelic frequency, %	DQB1	Allelic frequency,%
*01:01	4.2	*01:01	3.5	*02:01	13.6
*01:02	0.3	*01:02	10.2	*02:02	6.3
*03:01	10.0	*01:03	9.6	*02:03	1.3
*03:02	0.3	*01:04	9.0	*02:04	2.6
*03:07	0.6	*02:01	9.6	*03:01	17.6
*03:12	0.3	*03:01	13.1	*03:02	7.5
*03:16	1.9	*03:02	8.3	*03:03	2.6
*03:29-57	0.6	*03:03	5.1	*03:04-22	11.7
*04:01	3.8	*04:01	2.6	*04:01	0.6
*04:02	0.6	*04:02-04	0.3	*04:02	2.6
*04:03	0.9	*05:01	9.6	*05:01	7.9
*04:04-32	2.8	*05:02	0.6	*05:02	4.8
*07:01	13.1	*05:03	1.0	*05:03	5.1
*08:01	0.9	*05:05	9.0	*05:04	1.0
*08:02	0.6	*05:06-10	5.7	*05:05	0.3
*08:03	1.6	*06:01	2.6	*06:01	2.6
*08:04-24	0.6	*06:02	0.3	*06:02	5.1
*09:01	4.5			*06:03	5.1
*09:02	0.3			*06:04-39	1.6
*10:01	1.2				
*11:01	2.2				
*11:02	0.6				
*11:03	2.8				
*11:04	3.8				
*11:06-62	2.5				
*12:01	1.9				
*12:02	0.6				
*13:01	8.6				
*13:02	2.9				
*13:03-103	5.7				
*14:01	4.9				
*14:03	1.9				
*14:04	1.9				
*14:05-72	4.2				
*15:01	5.1				
*15:02	1.2				
*16:01	0.6				

**Table 3:** Allelic frequency of HLA-DRB1 in the Kazakh population (Tarbagatay).

DRB1	Allelic frequency, %	DRB1	Allelic frequency, %	DRB1	Allelic frequency,%
*01:01	5.4	*07:01	1.9	*12:02-08	4.1
*01:02	1.3	*08:01	1.6	*13:01	8.0
*03:01	7.6	*08:02	1.9	*13:02	3.8
*03:02	0.6	*08:03	1.9	*13:03-103	4.8
*03:07	1.6	*08:04-24	3.5	*14:01	1.9
*03:12	0.6	*09:01	4.8	*14:03	1.6
*03:16	0.3	*09:02	1.3	*14:04	0.3
*03:29-57	2.5	*10:01	1.3	*14:05-72	7.1
*04:01	3.0	*11:01	3.5	*15:01	7.6
*04:02	1.0	*11:04	4.1	*15:02-05	3.0
*04:03	0.6	*11:06-62	2.5	*16:01	0.3
*04:04-32	2.2	*12:01	2.5		

### HLA Haplotypes

Three-loci haplotypes MHC Class II (DRB1-DQB1-DQA1 Table 4) and two-loci (DRB1-DQB1, DQB1-DQA1; Table 5) were constructed using Arlequin v3.11. The most haplotype frequencies in the Kazakh population (Astana) were also observed in a majority of the European and Asian populations. All datasets are available from the worldwide population allele frequency database [3]. This database was used for comparison of HLA data of worldwide populations. HLA data refers to the original inhabitants of these regions. The five most common DRB1-DQA1-DQB1 haplotypes in the Kazakh population were found to be DRB1*07:01-DQA1*02:01-DQB1*02:01/02:02 (8.0%), DRB1*03:01-DQA1*05:01-DQB1*02:01 (3.4%), DRB1*13:01-DQA1*01:03-DQB1*06:03 (2.9%), DRB1*14:01-DQA1*01:04-DQB1*05:02 (2.9%), and DRB1*13:01-DQA1*03:01-DQB1*03:01 (1.6%). Three-locus haplotypes with ≥ 1.0% frequencies in the Kazakh population are presented in Table 4 and are compared with different populations from around the world, which have been identified by their DRB1-DQB1-DQA1 haplotypes. Evaluation of haplotype frequency and linkage-disequilibrium (LD) parameters of HLA two-loci haplotypes (DRB1-DQB1, DQB1-DQA1) in the Kazakhs were estimated, and shown in Table 5.

**Table 4:** The Most frequent of DRB1-DQA1-DQB1 extended haplotypes and their frequencies in the Kazakh population (Astana).

Haplotypes	HF, %	HF in the World	Possible Origin
DRB1*07:01-DQA1*02:01-DQB1*02:01/02:02^a^	8.0	very common	European/Asian
DRB1*03:01-DQA1*05:01-DQB1*02:01^b^	3.4	very common	European/Asian
DRB1*13:01-DQA1*01:03-DQB1*06:03^c^	2.9	very common	European/Asian
DRB1*14:01-DQA1*01:04-DQB1*05:02^d^	2.9	common	Asian
DRB1*13:01-DQA1*03:01-DQB1*03:01^e^	1.6	not found	Kazakhs
DRB1*15:01-DQA1*01:02-DQB1*06:02^f^	1.3	very common	European/Asian
DRB1*07:01-DQA1*03:01-DQB1*02:02^e^	1.3	not found	Kazakhs
DRB1*15:01-DQA1*01:02-DQB1*06:02^g^	1.3	very common	European
DRB1*07:01-DQA1*03:02-DQB1*02:02^e^	1.0	not found	Kazakhs
DRB1*15:01-DQA1*01:02-DQB1*06:03^h^	1.0	rare	European/Asian
DRB1*15:02-DQA1*01:03-DQB1*06:01^i^	1.0	very common	European/Asian
DRB1*03:01-DQA1*03:01-DQB1*03:02^e^	1.0	not found	Kazakhs
DRB1*09:01-DQA1*03:03-DQB1*02:02^e^	1.0	not found	Kazakhs
DRB1*14:03-DQA1*05:01-DQB1*03:01^j^	1.0	common	Asian
DRB1*13:01-DQA1*01:03-DQB1*06:04^k^	1.0	very rare	African
DRB1*13:02-DQA1*01:02-DQB1*06:04^l^	1.0	common	European/Asian
DRB1*04:01-DQA1*03:02-DQB1*03:01^e^	1.0	not found	Kazakhs
DRB1*13:01-DQA1*01:03-DQB1*05:01^e^	1.0	not found	Kazakhs

a Found in Buryats (22.0%); Khanty-Mansi (16.9%) Kazakhs (China) (8.3%).

b Found in Italians (Sardinia) (25.3%); Russia (North-west) (9.0%); Kazakhs (China) (13.1%).

c Found in Khanty-Mansi (8.1%); Italians (7.6%), Todzhinians (6.8%); Russia (North-west) (5.5%). Kazakhs (China) (4.8%);

d Found in South Korea (2.9%).

e Not found in any other population.

f Found in Todzhinians (22.5%); English (14.1%), Australia Aborigine (10.0%); Russia (North-west) (9.0%); Kazakhs (China) (2.4%).

g Found in Italians (14.1%); Slovenes (11.4%).

h Found in Australia Aborigine (7.0%).

i Found in Japan (8.2%); Mongolians (6.5%); Kazakhs (China) (2.4%).

j Found in Khanty-Mansi (8.1%); Italians (7.6%); Kazakhs (China) (4.8%).

k Found in Cameroon Yaounde (1.2%).

l Found in Italians (1.9%); Tuva (1.1%).

**Table 5:** Haplotype frequency and significant linkage disequilibrium parameter of HLA two-loci haplotypes in Kazakh population (Astana).

DRB1-DQB1	n^a^	HF^b^	D^c^	t^d^	DQB1-DQA1	n^a^	HF^b^	D^c^	t^d^
DRB1*07:01-DQB1*02:01	23	0.0732	0.4650	5.48	DQB1*02:01-DQA1*02:01	23	0.0732	0.7296	4.11
DRB1*03:01-DQB1*02:01	11	0.0350	0.2552	4.15	DQB1*03:02-DQA1*03:01	15	0.0478	0.5687	3.13
DRB1*07:01-DQB1*02:02	10	0.0318	0.4249	2.61	DQB1*03:01-DQA1*05:01	15	0.0478	0.3914	5.35
DRB1*13:03-103-DQB1*03:01	7	0.0223	0.2562	3.21	DQB1*03:01-DQA1*05:05	12	0.0382	0.3045	4.99
DRB1*11:04-DQB1*03:01	6	0.0191	0.3915	2.14	DQB1*02:01-DQA1*05:01	10	0.0318	0.2275	4.11
DRB1*14:05-72-DQB1*03:01	6	0.0191	0.3446	2.32	DQB1*03:04-22-DQA1*03:02	8	0.0254	0.2123	3.15
DRB1*03:01-DQB1*03:02	4	0.0127	0.0754	2.37	DQB1*05:01-DQA1*01:04	7	0.0223	0.2095	2.23
DRB1*13:01-DQB1*03:01	4	0.0127	-0.1693	4.82	DQB1*03:01-DQA1*05:06-10	7	0.0223	0.2562	3.21
					DQB1*03:04-22-DQA1*05:05	6	0.0191	0.1061	3.39
					DQB1*03:04-22-DQA1*03:01	6	0.0191	0.0314	4.96
					DQB1*03:01-DQA1*03:01	6	0.0191	-0.1794	7.31
					DQB1*03:04-22-DQA1*05:06-10	5	0.0159	0.1783	2.18
					DQB1*05:01-DQA1*01:02	4	0.0127	0.0647	2.55
					DQB1*03:01-DQA1*03:02	4	0.0127	-0.1373	4.64

a Number of times;

b Haplotype frequency;

c D Linkage disequilibrium;

d Only t values ≥ 2.0 were considered significant.

### Neighbor-Joining Dendrogram

The Neighbor-joining dendrogramm was created using the allelic frequencies at the HLA-DRB1 locus of various populations including the Kazakh group (Fig. 2). DRB1 allele frequencies between the Kazakh population and other world populations (European, Scandinavian, Mediteranean, Siberian and Asian populations) were compared. Results showed a clear divergence among these world populations. The genetic distance dendrogram (Fig. 2) shows that the Kazakh population is clustered together with Asian and Siberian populations, separate from European, Scandinavian and Mediterranean populations. The genetic structure of the Kazakhs is therefore shown to be closest to the Asian and Siberian populations.

### Multidimensional Scaling Analysis

**Figure 3:**
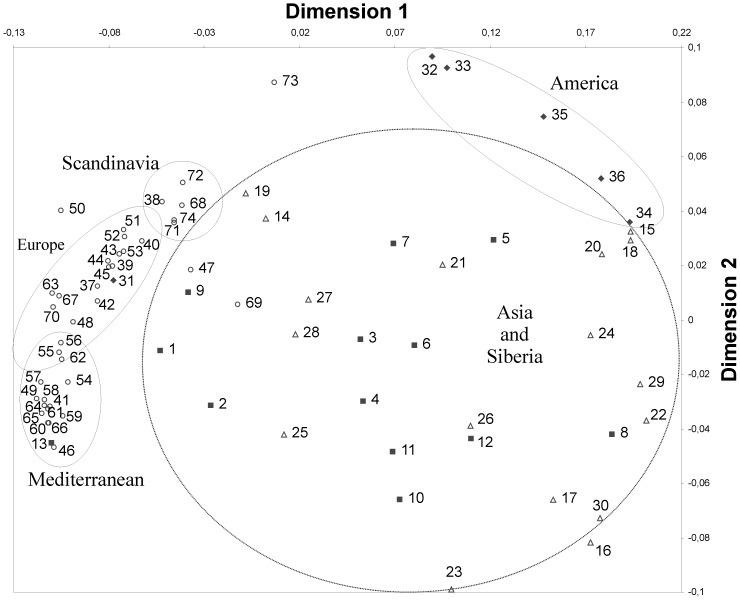
Multidimensional scaling analysis (MDS) of 74 populations tested for the HLA-DRB1 polymorphism. Each point represents a population and its symbol its geographic region: ▪ - Asia 1-13 (1 Kazakhs (Astana), 2 Kazakhs (Tarbagatay), 3 Mongolian, 4 Han Chinese, 5 Japanese, 6 Kazakhs (China), 7 Koreans, 8 Taiwanese, 9 Uyghurs, 10 Malay, 11 Thai, 12 Vietnamese, 13 Turkish); Δ - Siberia 14–30 (14 Aleuts, 15 Chukchi, 16 Evenks, 17 Кеt, 18 Koryaks, 19 Buryats, 20 Nedigal, 21 Nentsy, 22 Nivkhs, 23 Nganasan, 24 Evenki (Okhotsk), 25 Todzhinians, 26 Tofalar, 27 Tuvinians-1, 28 Tuvinians-2, 29 Udegeys, 30 Ulchi); ♦ - America 31–36 (31 Argetine, 32 Mazatecans, 33 Ache, 34 Eskimos-1, 35 Eskimos-2, 36 Ypika_Alaska); ○ - Europe 37–74 (37 Austrians, 38 English, 39 German, 40, French_West, 41 Italians (North Italy), 42 Netherlands, 43 Portuguese, 44 Spanish (Granada), 45 Spanish (Madrid), 46 Albanians, 47 Bashkirs, 48 Belarussian, 49 Bulgarian, 50 Chuvashians, 51 Polish, 52 Russian (North-West), 53 Russian (Ural), 54 Serbs, 55 Slovaks, 56 Ukranian, 57 Armenians, 58 Cretans, 59 Georgians, 60 Greece, 61 Italians (South Italy), 62 Arabs, 63 Jews_Ashkenazi, 64 Kurds, 65 Lebanese, 66 Macedonians, 67 Palestinians, 68 Finnish, 69 Khanty-Mansi, 70 Komis, 71 Norway, 72 Pomors, 73 Saami, 74 Swedish). Stress value  =  0.10.

Because of the multiethnic background of the Kazakh population, multidimensional scaling analysis for the Kazakhs with different worldwide populations was performed. Multidimensional scaling analysis of the 74 ethnic groups were based on the allelic frequencies of the HLA-DRB1 locus shown in Fig. 3. The results show that all the ethnic groups can be divided into five clusters; Asian and Siberian, American, Scandinavian, European and Mediterranean populations. The results reveal that on the basis of the HLA-system, the Kazakhs are related to the Kazakhs from China, Uyghurs, Mongolians, Todzhinians, Tuvinians as well as to other Siberians and Asians.

## Discussion

This study aimed to determine the HLA class II (DRB1, DQA1 and DQB1) highly specific Kazakh alleles and specific HLA haplotypes, which have a low frequency in other world populations. The Kazakh's HLA alleles have been used for calculations of two-dimensional genetic distances, neighbor-joining dendrogramms, multidimensional scaling analysis (MDS), and the generation of extended HLA haplotypes (see Tables 4, 5). High frequencies of DRB1 *07:01 (13.1%) and DRB1*03:01 (10.0%) were found in the Kazakh population and similar frequencies of these two alleles were observed in other populations of Kazakhs [37]. DRB1*03:01 is present in almost all European and Asian populations [54], specifically in 13.1% Kazakhs (China) [37] and 14% Uyghurs [38]. DRB1*07:01 is presenting in almost all world populations, for example - in Germans 12.6%, in Russians (in 13.5% Ural [9], 12% in North-West Russians [14]), 10.7% in Kazakhs (China) [37] and 16.7% in Uyghurs [38], and 26% in Buryat [25]. The Frequencies of other European alleles were also reflected in the Kazakh population. Other more frequently seen alleles include the DRBl*13:01 allele (8.6%), which was much more common among Asian and Siberian populations, specifically Kets (23.5%) [31], Khanty Mansi (12.5%) [25], and Todzhinians (9.1%) [25]. Thus, DRB1*13:01 is present more in Siberian and Asian populations, than in European populations [63]. Typical asian alleles such as DRB1*09:01 (4.5%) are relatively frequent in Kazakhs. According to literature this allele is observed in Asians including up to 11.9% in Chinese [34] and 32% in Malasians [64]. It is interesting that in the Kazakhs, who live in Aktobe (Kazakhstan) this allele has a frequency of 3.9% [65], whereas the Kazakhs who live in Tarbagatay nearer to China, the frequency of this allele is 4.8%, while the Kazakhs who live in China have a frequency of 7.1% [37]. In contrast, the Chinese have a frequency of 11.9% [34] and Mongolians, 6.5% [3]. Thus, there is a tendency for the frequenty of alleles DRB1*09:01 to increase in Kazakhs going from west to east. European and Asian alleles are clearly presented in the Kazakh population. In Kazakhs from Astana, DRB1*04:05 was observed, which is more common in Asian populations with a frequency of 6–14% [63]. This observation is also made of populations of Mongolian origin. DRB1*11:01 and DRB1*11:04 alleles are rarer for native Arabian populations [3]. These two alleles were found in Kazakhs with frequencies of 2.2% and 3.8% respectively. With regard to HLA-DQB1, 19 alleles were found in the Kazakhs (Table 2). Two alleles exhibit frequencies higher than 30% in the Kazakh population, DQB1*02:01 (13.6%) and DQB1*03:01 (17.6%). DQB1*02:01 is also observed in the other Asian populations tested so far: Kazakhs (China) (23.8%) [37], Mongolians (11.5%) [3], Todzhinians (4.5%) [25] and Tuvinians from Russia (10.2%) [25], [33]. The DQB1*03:01 allele is slightly lower in Kazakhs (Astana), than in those related populations: Kazakhs (China) (21.4%) [37], Mongolians (25.5%) [3], Todzhinians (36.4%) [25] and Tuvinians (28.4%) [25], [33]. Several haplotypes were found and might be unique to Kazakhs, such as DRB1*07:01-DQA1*03:02-DQB1*02:02; DRB1*13:01-DQA1*01:03-DQB1*05:01 and DRB1*03:01-DQA1*03:01-DQB1*03:02, which were not found in any other world populations (Table 4). Haplotype frequency and significant linkage disequilibrium of HLA two loci haplotypes were identified in the Kazakh population and are shown in Table 5. The most common two loci haplotypes of DRB1-DQB1 in the Kazakhs with a frequency of more than 3% are DRB1*07:01-DQB1*02:01, DRB1*03:01-DQB1*02:01, DRB1*07:01-DQB1*02:02 (Table 5). The distribution of the most commom haplotype DQB1*02:01-DQA1*02:01 has a frequency of 7.3%.

The study polymorphism of mitochondrial DNAdata (Berezina G, 2011) shows that Western Europe (55%) and Eastern Europe (41%) mtDNA linkages are present in the Kazakh population. It has been indicated that a high degree of intensity of gene exchange has occurred between the Kazakh population and populations of Russia on the North-West, North, North-East and East of Kazakhstan (Berezina G, 2011). It was also supported, that Kazakh Y-chromosome markers belong largely to the C3*, C3c and O3 haplogroups, which were obtained from people of southern Siberian or Mongolian lineage [66]. The highest frequencies of the C3* star-cluster (from 3 to 30%) were observed in Altaian Kazakhs [67], known as the C3* star-cluster ascribed to the descendants of Genghis Khan. Frequencies of Haplogroup C are very common in Mongolia (15%) and in populations of Central Asia (7–18%) [68].

In 1991, when the study of HLA allelic diversity was conducted few Caucasoid, Mongolian and mixed ethnic groups living in the territory of the former USSR were chosen. Based on these results, several authors concluded that the data on HLA-markers was broadly consistent with the anthropological information [69]. Kazakhs are characterized by the presence of HLA alleles that are also present in Caucasians and in Asians, although each of these populations has its own particular HLA- profile. The distribution of HLA alleles in Kazakhs agrees broadly with similar data for populations from Mongolia. This is confirming a hypothesis of the existence of gene flow between European, Asian and Siberian peoples, and may be due to the migration of peoples from Asia and/or Siberia into Europe. Cultural features of people in Eurasia corroborate genetic contacts between Asia and Siberia. These results could suggest that Kazakhs were genetically admixed with Caucasian, Siberian and Asian populations. Kazakhs, Uyghurs, Buryats, Mongolians and northern China inhabitants are representing a certain intermediate group, which is gradually loosing the HLA-specificities characteristic of the European groups and accumulating HLA-alleles specific to south-east Asian populations [70], [71].

The date of the HLA class II neighbor-joining tree shows the relatedness of world populations with the Kazakh population (Fig. 3). Populations are grouped in two main branches which are related. On on side are clustered Kazakhs (Astana and Tarbagatay), Asians and Siberians, Chinese Kazakhs, Tuvinians, and Todzhinians. On the other side are grouped European, Mediterranean, and Scandanavian ethnic groups. The Kazakh population (Astana) shows the closest genetic relation with Siberians and Asians. The study on HLA polymorphisms, which includes historical and genetic data support that the Kazakh population is characterized by the features of the Central Asian anthropological type under the influence of different groups such as Asian, Siberian, and European anthropological types. Migrations and mixing of many different ethnic groups are the major factor determining the genetic diversity of Kazakh population. Finally, Kazakhs are genetically different from other Asians (Figs. 2 and 3) as their HLA genetic pool has alleles from European, Asian and Siberian populations. Kazakhs (Astana) are related to Kazakhs (Tarbagatay), also to Kazakhs (China) and Uyghur groups (Fig. 3). A genetic distance-based analysis clustered the populations into groups according to their geographic origin. The structure of genetic variation of the Kazakh population tended to have distinct geographic occurrences, in agreement with the distance clusters.

It should be noted that the relatively high degree of heterogeneity in Asian and Siberian populations compared to European populations may be associated with a wider habitat residence. Asian, especially the Siberian peoples, are relatively isolated from each other, whereas European populations are living in a more compact and limited area, providing more intense interactions. Previous studies and current results support a unique genetic origin of the Kazakhs, and this population could be genetically an admixture of three ethnic groups: Europeans, Siberians and Asians. Our results suggest that HLA loci and haplotypes in the Kazakh population are significant genetic polymorphisms, that will allow a future use of our results to find an HLA-matched donor, specifically for bone marrow transplantation, which in turn suggests the clinical relevance of ours and future research in the Kazakh population. Such studies are in high demand, as the data in this region is very limited. These data can be used for any research into HLA and disease, specifically relevant is data that has already been used in the study of tuberculosis in the Kazakh population [72].
